# RNA-sequencing of the sturgeon *Acipenser baeri* provides insights into expression dynamics of morphogenic differentiation and developmental regulatory genes in early versus late developmental stages

**DOI:** 10.1186/s12864-016-2839-3

**Published:** 2016-08-08

**Authors:** Wei Song, Keji Jiang, Fengying Zhang, Yu Lin, Lingbo Ma

**Affiliations:** 1 Key Laboratory of East China Sea and Oceanic Fishery Resources Exploitation and Utilization, Ministry of Agriculture, Shanghai, 200090 China; 2 East China Sea fisheries Research Institute, Chinese Academy of Fishery Sciences, Shanghai, 200090 China

**Keywords:** *Acipenser baeri*, Transcriptome sequencing, Differentially expressed genes, Embryonic stages, Late stages, Development

## Abstract

**Background:**

*Acipenser baeri*, one of the critically endangered animals on the verge of extinction, is a key species for evolutionary, developmental, physiology and conservation studies and a standout amongst the most important food products worldwide. Though the transcriptome of the early development of *A. baeri* has been published recently, the transcriptome changes occurring in the transition from embryonic to late stages are still unknown. The aim of this work was to analyze the transcriptomes of embryonic and post-embryonic stages of *A. baeri* and identify differentially expressed genes (DEGs) and their expression patterns using mRNA collected from specimens at big yolk plug, wide neural plate and 64 day old sturgeon developmental stages for RNA-Seq.

**Results:**

The paired-end sequencing of the transcriptome of samples of *A. baeri* collected at two early (big yolk plug (T1, 32 h after fertilization) and wide neural plate formation (T2, 45 h after fertilization)) and one late (T22, 64 day old sturgeon) developmental stages using Illumina Hiseq2000 platform generated 64039846, 64635214 and 75293762 clean paired-end reads for T1, T2 and T22, respectively. After quality control, the sequencing reads were *de novo* assembled to generate a set of 149,265 unigenes with N50 value of 1277 bp. Functional annotation indicated that a substantial number of these unigenes had significant similarity with proteins in public databases. Differential expression profiling allowed the identification of 2789, 12,819 and 10,824 DEGs from the respective T1 vs. T2, T1 vs. T22 and T2 vs. T22 comparisons. High correlation of DEGs’ features was recorded among early stages while significant divergences were observed when comparing the late stage with early stages. GO and KEGG enrichment analyses revealed the biological processes, cellular component, molecular functions and metabolic pathways associated with identified DEGs. The qRT-PCR performed for candidate genes in specimens confirmed the validity of the RNA-seq data.

**Conclusions:**

This study presents, for the first time, an extensive overview of RNA-Seq based characterization of the early and post-embryonic developmental transcriptomes of *A. baeri* and provided 149,265 gene sequences that will be potentially valuable for future molecular and genetic studies in *A. baeri.*

**Electronic supplementary material:**

The online version of this article (doi:10.1186/s12864-016-2839-3) contains supplementary material, which is available to authorized users.

## Background

During metazoan embryonic development, a totipotent zygote divides, grows and experiences intense post-embryonic anatomical and physiological changes resulting in an adult living organism constituted of different specific tissues [1]. This process is naturally correlated with spatial or temporal changes in gene expression. Therefore, complete assessment of gene expression levels during ontogeny of an organism would be fundamental for giving a comprehensive dataset for genetic regulation of developmental processes. Up to date, a large body of literature has reported the use of in situ hybridization, microarrays, and more latterly transcriptome sequencing (RNA-seq) technologies for profiling gene expression during development of various model and non-model organisms [1–6]. Sturgeons, one of the earliest origins of vertebrate groups, constitute an important archetypal material for studying the origin of species and evolution [7–9]. Moreover, sturgeons are listed in the appendix of the endangered species by the Convention on International Trade in Endangered Species of wild fauna and flora (CITES) [10]. However, scientific studies on molecular mechanisms controlling the development of sturgeons are scarce and generally focused on a narrow range of single genes or gene sets. Transcriptomics resources for sturgeons have emerged just recently and, until now, data of sturgeon transcriptome include those made available owing to recent studies on reproductive tissues [11, 12] obtained by next-generation pyrosequencing of gonad transcriptomes of *Acipenser fulvescens, de novo* assembly of the gonadal transcriptome of *Acipenser sinensis* and microRNA transcriptome and expression assay in *Acipenser schrenckii* [13]. In addition, we have recently made available the largest sturgeons’ transcriptomics data using RNA-sequencing (RNA-seq) to generate the transcriptome for the early development of *A. baeri* [14]. Nevertheless, little is known about late developmental stages of *A. baeri* and in regards to the molecular background concerning the transition from pre-larval to juvenile stages, even less information has been made accessible, thus hindering aquaculture practices for this species. Studying this undiscovered molecular areas of *A. baeri*’s developmental biology, especially gene regulation underlying the transformation of embryos into adult fish, would be vital for feeding, reproductive and fish health management purposes, and would give insights into the biology of sturgeons and other related fish species.

The aim of the present study was to assess the transcriptome and the gene expression dynamics of three developmental stages of *A. baeri* ranging from the embryonic up to the 64 days old sturgeon stages.

## Results

### Sequencing data quality assessment and *de novo* assembly

In the present research, we utilized samples of *A. baeri* collected at three specific developmental stages including big yolk plug (T1, 32 h after fertilization), wide neural plate formation (T2, 45 h after fertilization) and 64 day old sturgeon with electric sensors ganglion (T22). RNA Sequencing via the Illumina HiSeq2000 system (Table 1) produced about 64109484, 64708472 and 75356474 raw reads for T1, T2 and T22 covering 6.41, 6.47 and 7.54 Gb of sequence data, respectively. Over 90 % of the raw reads survived quality cleaning and trimming and resulted in 64039846, 64635214 and 75293762 clean reads respectively for T1, T2 and T22 with average length of 99.5 for T1 and T2, and 99.6 for T22. The sequencing reads were deposited in the NCBI Short Read Archive (SRA) database (http://www.ncbi.nlm.nih.gov/sra/) under the accession number SRP053165. The sequenced reads were assembled using *de novo* assembly method. After removal of transcripts with short open reading frames (ORFs) (<50 amino acids) and weakly supported transcripts or isoforms (mapped reads < 1 %), the final transcriptome contained 149,265 assembled unigenes with N50 value of 1277 bp (Additional files 1 and 2). Samples T1, T2 and T22 individually produced 81,450, 112,382 and 77,018 unigenes with mean lengths of 329.1046, 329.1957 and 327.6552 bp.

**Table 1:** Statistical results of raw and preprocessed sequences

Sample name	T1	T2	T22
Raw reads	64109484	64708472	75356474
Raw Gb	6410948400	6470847200	7535647400
Clean reads	64039846	64635214	75293762
Clean Gb	6372389027	6433659221	7498582876
Average length	99.5	99.5	99.6
Isoforms	136941	183065	439158
Unigenes	81448	112382	278166

### Functional annotation of unigenes

To determine the function of *de novo* assembled transcripts, the whole set of sequences were aligned against the NCBI Uniprot protein databases using BLASTX with an E-value cutoff of 1E-3. The significant alignment results are reported in Additional file 3. The result showed that 57,346 unigenes (38.42 %) had noteworthy hits to Uniprot databases equivalent to 45,837 single known proteins and 11,509 homologous orthology clusters in Uniprot protein databases whereas the remaining 61.58 % unigenes could stand for UTRs, non-protein coding genes or *A. baeri*-specific genes which were too different to be annotated by homology search with the adopted E-value cutoff. We performed analysis of BLASTX results to determine best species hits (Additional file 4). The results showed the hits of *A. baeri*’s transcripts with 1312 distinct species including fish species such as *Latimeria chalumnae* (5940 transcripts)*, Danio rerio* (4943 transcripts)*, Helobdella robusta* (4591 transcripts) and *Oreochromis niloticus* (2325 transcripts) and in minor extent to other vertebrate species.

The GO annotation was performed by the mapping of unigene sequences with Uniprot database and the recovery of GO terms linked to protein sequences in Uniprot. The results (Additional file 5) showed that a total of 43,062 unigenes (28.85 %) were ascribed at least one GO term in the GO classes of “cellular component”, “biological process” and “molecular function”.

In the KEGG database (Additional file 6), 29,526 unigenes (19.78 %) were annotated into 329 pathways. “Metabolic pathways” (2010 transcripts) was the major pathway and was accompanied by “biosynthesis of secondary metabolites” (642 transcripts) and “microbial metabolism in diverse environments” (417 transcripts). In the pathway class of signal transduction, “PI3K-Akt signaling pathway” (347 transcripts) was the most exemplified. Other pathways related to translation and diseases were similarly present. Overall, 27,773 unigenes were annotated in both GO and KEGG databases.

Interproscan annotation (Additional file 7) was exploited to detect conserved domains associated with the protein sequences. A total of 16,735 unigenes (11.21 %) were annotated in Interproscan and produced 6751 domains. The statistics (Additional file 8) revealed “zinc finger, C_2_H_2_” as the main conserved domain (count = 3392) followed by “WD40 repeat” (count = 2312) and “zinc finger, C_2_H_2_-like” (count = 1799).

### Unigene differential expression profiling

**Fig. 1:**
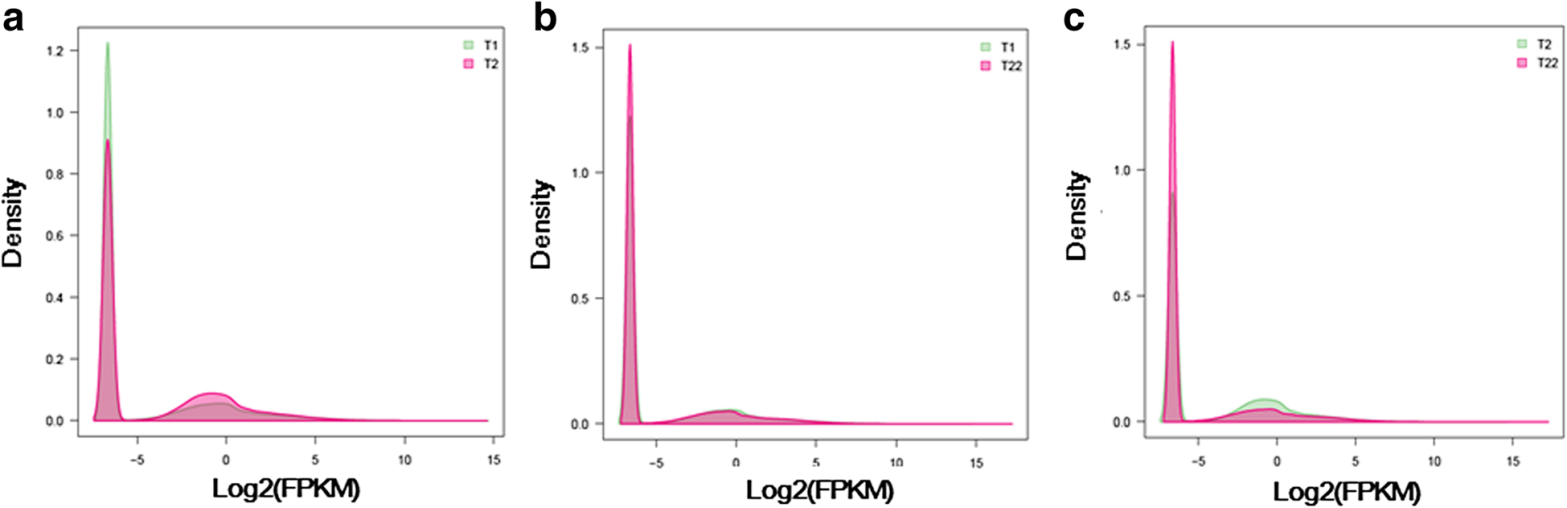
FPKM density of unigenes obtained by pairwise comparison of samples collected at big yolk plug (T1), wide neural plate formation (T2) and 64 day old (T22) stages of *A. baeri*. **a** FPKM density of unigenes by comparison of T1 vs. T2, **b** FPKM density of unigenes by comparison of T1 vs. T22, **c** FPKM density of unigenes by comparison of T2 vs. T22

The expression level of each unigene was estimated in FPKM units using the RSEM software protocol. After filtering transcripts with FPKM = 0 in at least one sample, a total of 37,045 unigenes were considered as expressed within the three samples (Additional file 9). The comparison of FPKM density of unigenes between samples is reported in Fig. 1.

**Fig. 2:**
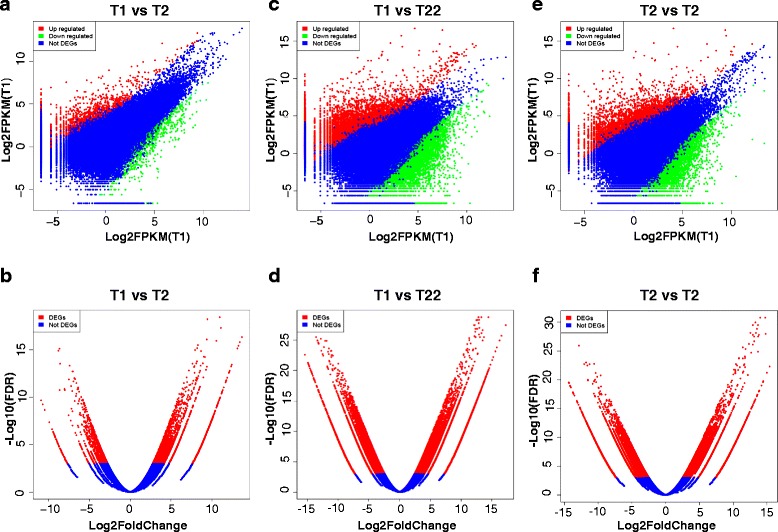
FPKM scatter plots and the Volcano plots obtained from pairwise comparison between samples collected at big yolk plug (T1), wide neural plate formation (T2) and 64 day (T22) old stages of *A. baeri*. The scatter plots compared the gene expression levels pairwise among the three libraries while Volcano plots were generated using log2 fold-change against -log10 (*p*-value) displaying the amount of differentially expressed genes (red dots). **a** FPKM scatter plot of unigenes obtained by pairwise comparison of T1 vs. T2, **b** Volcano plot of unigenes obtained by pairwise comparison of T1 vs. T2, **c** FPKM scatter plot of unigenes obtained by pairwise comparison of T1 vs. T22, **d** Volcano plot of unigenes obtained by pairwise comparison of T1 vs. T22, **e** FPKM scatter plot of unigenes obtained by pairwise comparison of T2 vs. T22, **f** Volcano plot of unigenes obtained by pairwise comparison of T2 vs. T22

**Fig. 3:**
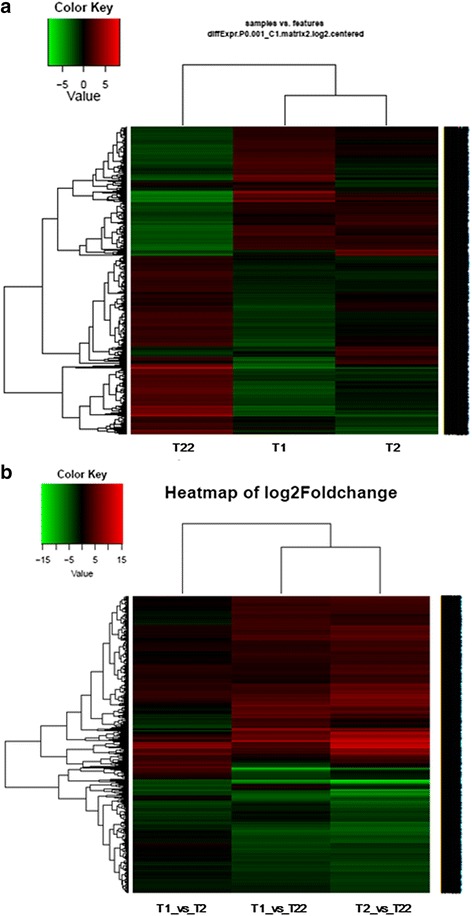
Expression pattern of DEGs screened between samples collected at big yolk plug (T1), wide neural plate formation (T2) and 64 day (T22) stages of *A. baeri*. **a** Heatmap of DEGs between samples based on FPKM units; the color key represents FPKM normalized log2 transformed counts and each row represents a gene. **b** heatmap of Log2(foldchange) of DEGs screened by pairwise comparison of the three samples

To specifically identify genes that affect the development process, we analyzed gene expression changes for the 3 developmental stages using edgeR package [15] for pairwise comparison (Additional file 10). The FPKM scatter plots and the Volcano plots obtained from pairwise comparison between samples are depicted in Fig. 2. The lists of all differentially expressed genes (DEGs) screened from T1 vs. T2, T1 vs. T22 and T2 vs. T22 are summarized in Additional file 11. Only DEGs with *p*-value < 0.05 at a FDR < 0.001 and fold change > 2 were considered significant. The heat map showing the expression pattern of these DEGs and the heatmap of Log2(foldchange) were depicted in Fig. 3. The statistics showed 2789, 12,819 and 10,824 DEGs for the respective T1 vs. T2, T1 vs. T22 and T2 vs. T22 comparisons. The pairwise comparison between T1 and T2 samples showed that DEGs were characterized by the upregulation of 1690 and the downregulation of 1099 genes while 6221 downregulated and 6598 upregulated DEGs were recorded by comparing T22 and T1. The comparison between T22 and T2 led to 5343 downregulated and 5481 upregulated DEGs. As shown in Additional file 12, clustering of DEGs generated 12 subclusters with 3 of them presenting important characteristics. The subcluster_2 was constituted of 262 DEGs initially highly expressed in T1 but downregulated in T2 and T22, progressively. The 2310 genes of the subcluster_3 were initially lowly expressed in T1 stage but upregulated in T2 and further in T22 stage. The subcluster_11 included 107 genes exclusively upregulated at T2 and probably in charge of morphogenetic transformations leading to adult individual.

**Fig. 4:**
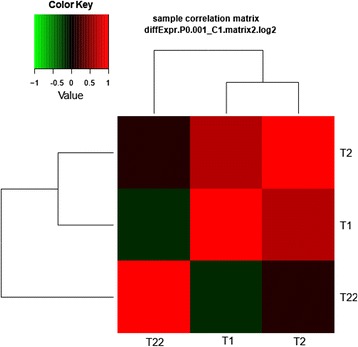
Analysis of sample correlation based on DEGs’ features between samples collected at big yolk plug (T1), wide neural plate formation (T2) and 64 day (T22) stages of *A. baeri*. There was a high correlation between T1 and T2 while no significant correlation was found between both samples and T22

The correlation analysis (Fig. 4) revealed that there was a positive correlation between T1 and T2 while a negative correlation was observed in T1 vs. T22 comparison. No significant correlation was recorded for T2 vs. T22.

### GO and KEGG pathway enrichment analysis of DEGs within contiguous development stages

Between stage T1 and stage T2, screened DEGs were significantly (*p*-value < 0.05, FDR < 0.05) enriched in 105 GO terms with 90 of them being categorized in “biological process”, 6 in “cellular component” and 9 in “molecular function” (Additional file 13, Fig. 5a). The GO class of “biological process” was widely represented by single-organism developmental process (129 DEGs), multicellular organismal development (119 DEGs), developmental process (129 DEGs) and anatomical structure development (113 DEGs). Cellular component was overrepresented by intermediate filament (10 DEGs), cytosolic part (12 DEGs), non-membrane-bounded organelle (106 DEGs), intracellular non-membrane-bounded organelle (106 DEGs) and cytosolic ribosome (6 DEGs) while protein binding (131 DEGs), structural molecule activity (44 DEGs), kinase binding (18 DEGs) and protein kinase binding (15 DEGs) were found as significant molecular functions.

**Fig. 5:**
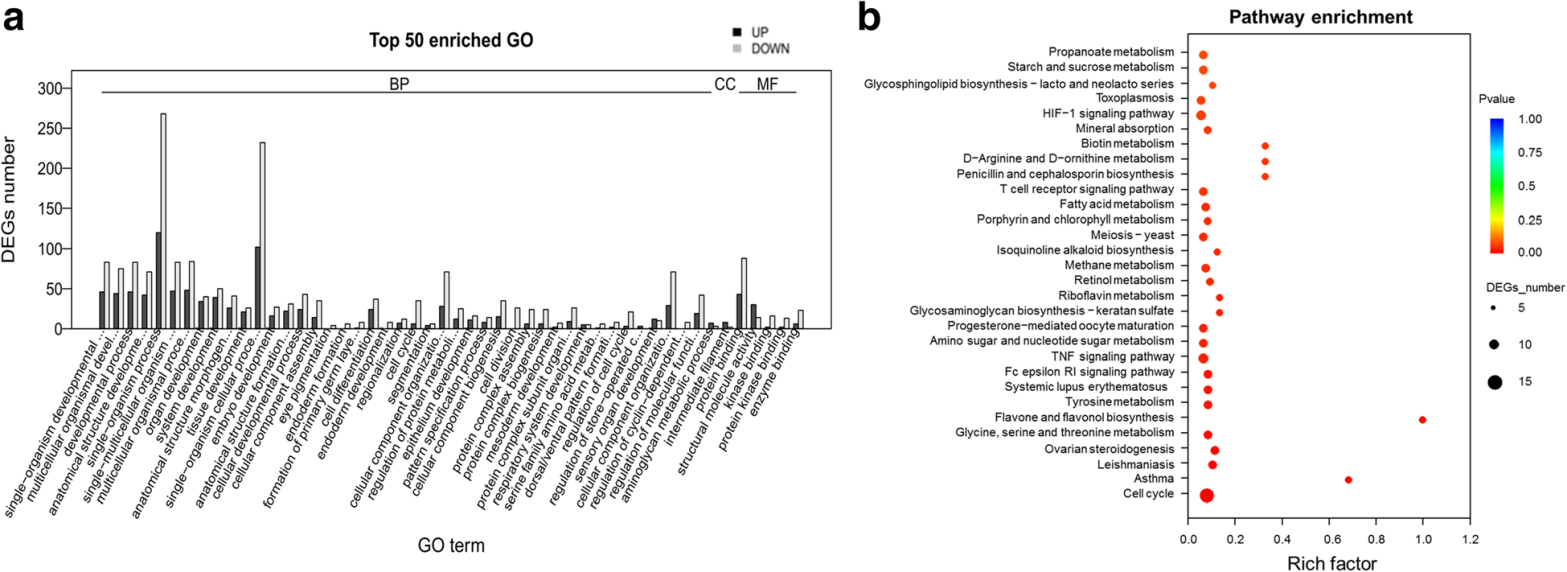
Functional enrichment analysis of DEGs screened between big yolk plug (T1) and wide neural plate formation (T2) stages of *A. baeri*. **a** Histogram of the most significantly enriched GO terms of DEGs screened between big yolk plug (T1) and wide neural plate formation (T2) stages of *A. baeri*. DEGs were enriched in 105 GO terms with 90 of them being categorized in “BP”, 6 in “CC” and 9 in “MF”. The X-axis represents the 50 most significant GO-terms in the three main GO categories: Biological process (BP), Molecular function (MF), Cellular component (CC), which were further separated into 44, 1 and 5 functional groups, respectively. The Y-axis represents numbers of downregulated and upregulated unigenes mapping to the given functional GO term. The significance of each GO term was estimated based on FDR corrected *p*-value (*p*-value < 0.05). **b** KEGG pathway enrichment of DEGs screened between big yolk plug (T1) and wide neural plate formation (T2) stages of *A. baeri*. The first 30 pathways were presented with 20 were significantly enriched. The X-axis represents rich factors of unigenes mapping to the given pathway. The Y-axis represents the 30 first pathways based on the decreasing order of *p*-values. The significance of each pathway was estimated based on FDR corrected *p*-values (*p*-value < 0.05)

In the KEGG pathway enrichment analysis (Additional file 14, Fig. 5b), the pathway class of “cell growth and death” with 12 downregulated unigenes participating in cell cycle was the most represented. Asthma (2 downregulated unigenes), leishmaniasis (1 upregulated and 4 downregulated unigenes) and ovarian steroidogenesis (1 upregulated and 3 downregulated unigenes) were the most significantly (*p*-value < 0.05) enriched pathways following cell cycle.

The GO enrichment of DEGs screened between T1 and T22 (Additional file 15, Fig. 6a), showed enrichment for 280 biological processes represented by multicellular RNA processing (111 DEGs among which 3 are upregulated), RNA metabolic process (384 DEGs encompassing 47 upregulated unigenes) and developmental process (344 DEGs including 81 upregulated unigenes), 63 cellular components represented by intracellular (1263 DEGs), organelle (985 DEGs), intracellular organelle (984 DEGs) and intracellular part (1162 DEGs), and 36 molecular functions exemplified by structural molecule activity (124 DEGs), structural constituent of ribosome (73 DEGs), transcription factor binding (45 DEGs), transcription factor binding transcription factor activity (44 DEGs). The most represented KEGG pathways among the 17 significantly (*p*-value < 0.05) enriched KO terms included transcriptional misregulation in cancer (21 unigenes) in the pathway class of “Cancers Overview”, ribosome biogenesis in eukaryotes (20 unigenes) and mRNA surveillance pathway (21 unigenes) in the pathway class of “Translation” (Additional file 16, Fig. 6b).

**Fig. 6:**
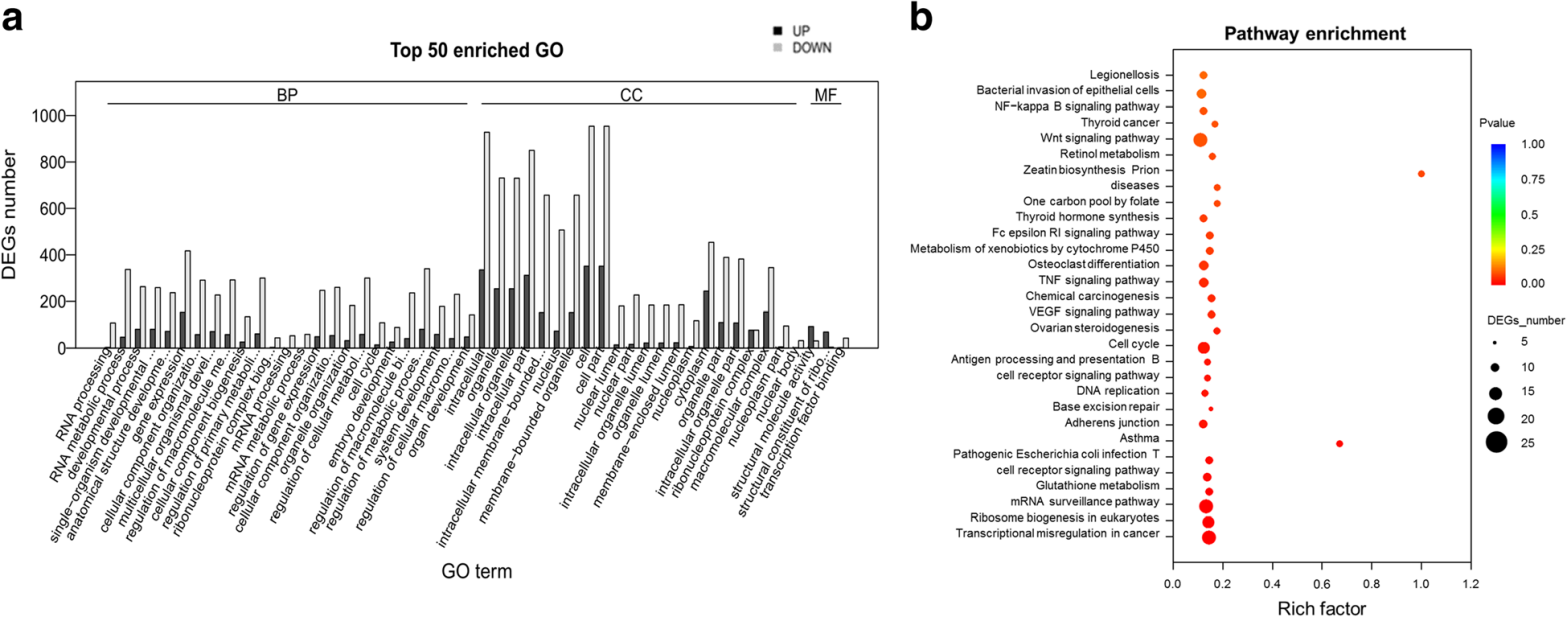
Functional enrichment analysis of DEGs screened between between big yolk plug (T1) and 64 day old (T22) stages of *A. baeri*. **a** Histogram of the most significantly enriched GO terms of DEGs screened between big yolk plug (T1) and 64 day old (T22) stages of *A. baeri*. DEGs were enriched in 379 GO terms with 280 of them being categorized in “BP”, 63 in “CC” and 36 in “MF”. The X-axis represents the 50 most significant GO-terms in the three main GO categories: Biological process (BP), Molecular function (MF), Cellular component (CC), which were further separated into 25, 22 and 3 functional groups, respectively. The Y-axis represents numbers of downregulated and upregulated unigenes mapping to the given functional GO term. The significance of each GO term was estimated based on FDR corrected *p*-values (*p*-value < 0.05). **b** KEGG pathway enrichment of DEGs screened between big yolk plug (T1) and 64 day old (T22) stages of *A. baeri*. The first 30 pathways were presented with 17 were significantly enriched. The X-axis represents rich factors of unigenes mapping to the given pathway. The Y-axis represents the 30 first pathways based on the decreasing order of *p*-values. The significance of each pathway was estimated based on FDR corrected *p*-values (*p*-value < 0.05)

The pairwise comparison between T2 and T22 allowed functional enrichment of DEGs in 273 GO terms (Additional file 17, Fig. 7a) and 28 KO pathways and (Additional file 18, Fig. 7b). For the GO category of “molecular function”, pathways significantly enriched were GTP binding (69 unigenes), transcription factor binding (31 unigenes) and guanyl nucleotide binding (84 unigenes) while developmental process (264 DEGs), anatomical structure development (239 DEGs) and single-organism developmental process (260 DEGs) were the most represented in the biological process category. Cellular component included membrane-bounded organelle (541 DEGs), nucleus (380 DEGs) and intracellular part (748 DEGs) as significantly enriched GO terms. Among the significant KO pathways, leishmaniasis (10 DEGs), ribosome biogenesis in eukaryotes (18 DEGs) and osteoclast differentiation (15 DEGs) pathways were the most dominant.

**Fig. 7:**
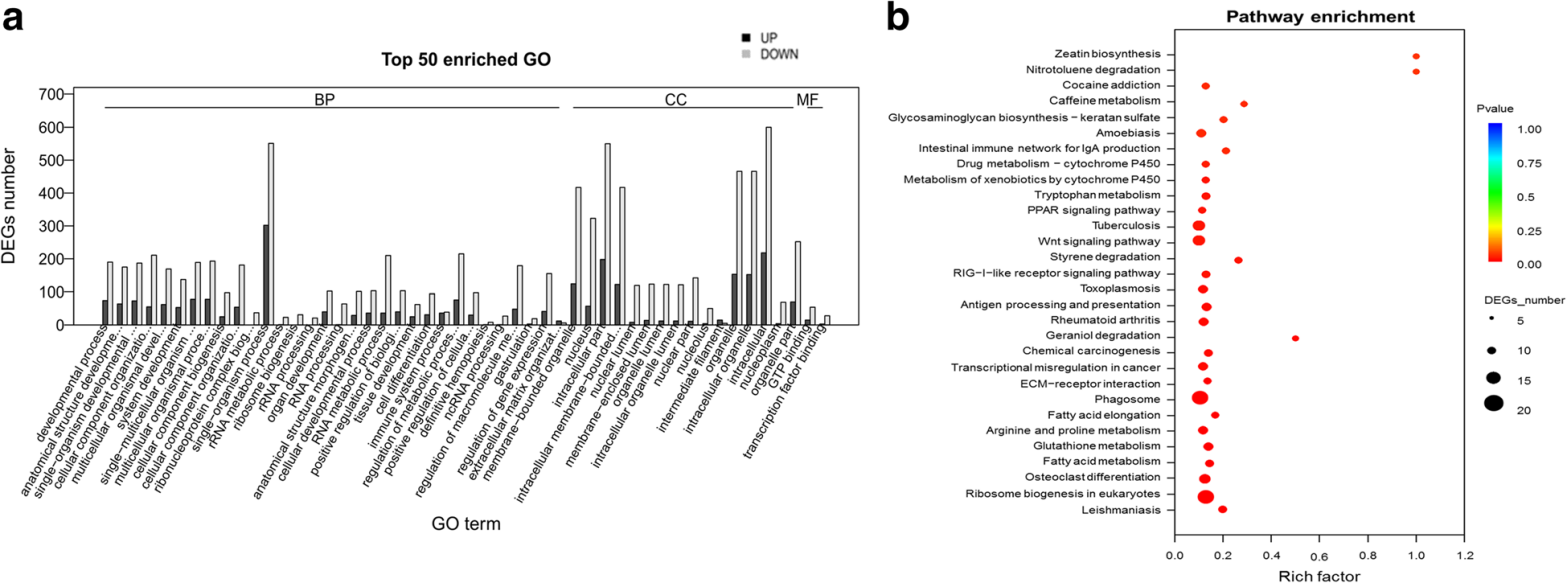
Functional enrichment analysis of DEGs screened between wide neural plate formation (T2) and 64 day old (T22) stages of *A. baeri*. **a** Histogram of the most significantly enriched GO terms of DEGs screened between wide neural plate formation (T2) and 64 day old (T22) stages of *A. baeri*. DEGs were enriched in 273 GO terms. The X-axis represents the 50 most significant GO-terms in the three main GO categories: Biological process (BP), Molecular function (MF), Cellular component (CC), which were further separated into 32, 16 and 2 functional groups, respectively. The Y-axis represents numbers of downregulated and upregulated unigenes mapping to the given functional GO term. The significance of each GO term was estimated based on FDR corrected *p*-values (*p*-value < 0.05). **b** KEGG pathway enrichment of DEGs screened between wide neural plate formation (T2) and 64 day old (T22) stages of *A. baeri*. The first 30 pathways were presented with 28 were significantly enriched. The X-axis represents rich factors of unigenes mapping to the given pathway. The Y-axis represents the 30 first pathways based on the decreasing order of *p*-values. The significance of each pathway was estimated based on FDR corrected *p*-values (*p*-value < 0.05)

### Study case 1: differences among morphogenic differentiation regulatory genes

In order to identify transcripts implicated in morphogenesis, we screened the GO terms by searching for terms in relation with morphogenesis using the GO enrichment file. On this basis, we found 260 DEGs associated with morphogenesis in the category of biological process. The GO terms associated with these DEGs in the three pairwise comparisons and their expression profiles are summarized in Additional file 19. Morphogenic differentiation DEGs screened between T1 and T2 were associated with the GO terms of anatomical structure morphogenesis, anatomical structure formation involved in morphogenesis, tissue morphogenesis, embryonic morphogenesis and morphogenesis of follicular epithelium. We observed that 38 of these DEGs including *ACTA1, twn-A, AANF, buc, dlx5a, NKD1, LOC770168, LOC101155136, APLNR, APOD, tbx16, LOC101164950, sox2* and *cbs* were identified as up-regulated unigenes while 35 unigenes including *bbs9, DUSP4, CABZ01041002.1, CDC42SE2, Ndp, robo4, robo4, ERRFI, LOC100722730, epha7, D623_10029598, epb41l5, LAMA1* and *CTNNB1* were downregulated. In the transition from T1 to T22, *ACTA1, twn-A, AANF, buc, dlx5a, NKD1, LOC770168, LOC101155136, APLNR, APOD, tbx16, LOC101164950* and *sox2* were found among the upregulated DEGs while the 166 downregulated DEGs included *robo4, ERRFI, LOC100722730, epha7, D623_10029598, epb41l5, LAMA1* and *CTNNB1.* These DEGs were associated with anatomical structure morphogenesis, embryonic morphogenesis, anatomical structure formation involved in morphogenesis, tissue morphogenesis, morphogenesis of an epithelium and blood vessel morphogenesis. The transition from T2 to T22 was characterized by the upregulation of 31 and the downregulation of 131 DEGs encoding for biological processes such as anatomical structure morphogenesis, blood vessel morphogenesis, anatomical structure formation involved in morphogenesis, cellular component morphogenesis, cell morphogenesis, embryonic morphogenesis, regulation of cell morphogenesis and regulation of anatomical structure morphogenesis. The highest number of morphogenic differentiation DEGs (213) was found in T1 vs. T22 comparison while the lowest one (73) was found between T1 and T2. The T2 vs. T22 comparison allowed the identification of 162 DEGs.

### Study case 2: changes among developmental regulatory genes

The definitive objective of this study was to identify an assortment of potential genes involved in developmental processes. Through analysis of GO enrichment output file, we found that 517 DEGs were directly implicated in developmental processes (Additional file 20). In T1 to T2 comparison, 151 DEGs were enriched in 22 developmental process including embryo development, organ development, system development and tissue development with *nos3, Schip1, fbn2b, LOC101073546* and *LOC100546827* as the most upregulated DEGs while the most downregulated DEGs included *birc5b, buc, LOC101155136, nanog, LSM14B, lft1* and *LOC101064479*. In the T1 vs. T22 analysis, 35 enriched GO terms (biological processes) were orchestrated by the expression of 416 DEGs. Genes *POSTN, f3a, LOC101073546, SCEL, COL1A3* and *gfra3* were found as the most upregulated whereas f*gfrl1b, twn-A, lft1, nanog, buc, LOC101064479* and *tbx16* were significantly downregulated. In the comparison of T2 and T22, 80 upregulated DEGs including *COL1A3, HG2A, LOC100534402, MBP, NEBL* and *alas2,* and 237 down-regulated DEGs containing *nanog, FOXP1B, LOC100709614, alcam,fgfrl1b, twn-A, RIPP1, buc* and *tbx16* were in charge of 28 biological processes*.* Similarities between T1 vs. T22 and T2 vs. T22 were observed, which further confirmed that T2 presented intermediary transcriptional changes.

### Candidate gene qRT-PCR validation

To validate the sequencing data, we randomly chose five unigenes, namely *six3a, sox17, HOXD10, wnt11b* and *eya3* genes, to perform the qRT-PCR experiment using the same pooled RNA employed for generating our RNA-seq data. The expression levels of each unigene obtained by RNA-seq or RT-PCR are presented in Additional file 21. In the sequencing result, we found that *six3a* was upregulated in T2 vs. T1 and T22 vs. T2 comparisons. *HOXD10* as well as *sox17* were upregulated in T2 compared to other samples. Initially highly expressed in T1, *wnt11b* and *eya3* were progressively downregulated until T22. The trends of expression of all five candidate genes, measured by qRT-PCR, were correlated with those of RNA-seq method. Altogether, qRT-PCR results largely supported RNA-seq results.

## Discussion

With the advent of next-generation sequencing and the development of bioinformatics systems [16], RNA sequencing, which is preferred compared with the conventional Sanger sequencing due to its low cost and high-throughput generation of quality transcriptome data, has turned into an essential instrument used in research and relatively short reads can be successfully assembled for non-model organisms [17–20]. In this study, we have produced the first broad map of *A. baeri* transcriptome using an Illumina paired end RNA-seq platform for inspecting gene expression dynamics in early and late developmental stages. Around 64039846, 64635214 and 75293762 high quality reads were generated respectively for T1, T2 and T22 from HiSeq 2000 and *de novo* assembled into 149,265 unigenes. The number of unigenes was higher than that reported in the testicular and ovarian transcriptomes of *A. schrenckii* containing respectively 122,381 and 114,527 unigenes [21] and that of the gonads transcriptome of *A. sinensis* constituted of 86,027 unigenes [22] or the 55,000 high quality ESTs of *A. naccarii* organized into a freely available AnaccariiBase [11, 23]. The higher number of unigenes generated here could be due to the fact that we performed sequencing using whole bodies of *A. baeri* samples, a deep sequencing coverage or differences in assembly softwares used. In addition, we remarked the increase of unigene amount in the transition from early developmental stages to subsequent late stages probably because of the activation of additional unigenes necessary for achieving new biological processes that arise harmoniously with the developmental course. This was in accordance with the fact that initial stages of vertebrate development depend dominatingly on maternal factors deposited in the egg with negligible zygotic translation until the complete activation of the embryonic genetic material [24, 25]. The validity of *de novo* assembly was assessed by examination of assembled unigenes using publicly available protein databases, functional annotation and validation of randomly selected unigenes by the qRT-PCR. Annotation of unigenes showed that 57,346 unigenes (38.42 %) had noteworthy homologs in the Uniprot databases while 28.85 % of unigenes were ascribed at least one GO term in the GO classes of “cellular component”, “biological process” and “molecular function”. In the KEGG database, 19.78 % of unigenes were annotated into 329 pathways. The Interproscan annotation produced 6751 domains corresponding to 16,735 unigenes (11.21 %). Though various technologically advanced *de novo* assemblers (e.g. Trans-AbySS, Oasis, SOAP2denovo and Trinity) have been established, scientific studies have proven the efficacy of Trinity since it is advantageous for annotation of transcriptomes for diverse vertebrate species [19, 26]. Here, a substantial amount of the Trinity *de novo* assembled unigenes was annotated in known protein databases. The unigenes without hits presumably fit in with untranslated regions, non-coding RNA, novel genes, short sequences without protein domain or assembly errors, and suggests that the *A. baeri* genome is still incomplete. Our study will help in the future completion and full annotation of the *A. baeri*’s genome.

We equally examined the differential expression of unigenes and identified 2789, 12,819 and 10,824 DEGs from T1 vs. T2, T1 vs. T22 and T2 vs. T22 comparisons according to our RNA-seq data and high correlation was found between T1 and T2 while no significant correlation was recorded for T1 vs. T22 and T2 vs. T22. This result showed that the DEGs match expectations in that the fewest number of differences were observed between the two early developmental stages (T1 vs. T2), and the greatest number was observed between T1 and T22, while T2 vs. T22 differences were intermediate. Based on GO enrichment analysis of DEGs, we identified 260 DEGs involved in morphogenic differentiation and 517 DEGs encoding for diverse developmental processes. Besides, the GO and KEGG pathway functional annotation of the overall set of DEGs allowed identification of multiple functions and pathways in which DEGs between samples were involved. These data give insight in the gene expression alterations occurring in the transition from the early stage to the late stage of *A.baeri* development.

Although the RNA-Seq experiment was performed using pooled RNA extracted from 3 specimens per development stage, the dataset presents some limitations. There were no biological or technical replicates for the pairwise comparison between the 3 samples. The dataset is therefore un-replicated and ‘sample’, but not really ‘development-stage’ specific. Consequently, although the statistical analysis of many transcripts yields statistically significant changes between these 3 pooled samples, the biological replicate is still *N* = 1, so there is no variation in gene expression addressed. In addition, the present RNASeq data is a transcriptome screen, not necessarily a “functional assay” and the fact that we did not perform some translational experiments could mislead our interpretation because the gene function is not necessarily determined using RNA-Seq given that, while it is regarded and experimentally proven that instances of up- or down-levels of expression generally translate (no pun intended) into higher or lower protein levels, respectively, this cannot be automatically assumed without further biochemical analyses. Post-translational modifications do occur that could alter anticipated final protein levels and hence influence pathway interactions.

## Conclusions

Our research established, for the first time, an extensive overview of RNA-Seq based characterization of the early and post-feeding developmental transcriptome of *A. baeri* as well as significant data on differential expression among both late and early developmental stages. The RNA-seq provided considerable gene sequences that will be valuable for future molecular and genetic studies in *A. baeri* and other related sturgeon and fish species.

## Methods

### Fish specimens, mRNA extraction and Illumina sequencing

In this study, we collected samples of *A. baeri* at three distinct developmental stages. Embryos were raised from inseminated eggs acquired from commercial contractors (Hangzhou Qiandaohu Xunlong Sci-tech Development Co. Ltd in China) and kept in fresh water aquaria that were maintained at 18–21 °C for 1 week to several months. The developmental stages selected for this study included big yolk plug (T1, 32 h after fertilization), wide neural plate formation (T1, 45 h after fertilization) and 64 day old sturgeon with electric sensors ganglion (T22). Because there are inter-individual differences in developmental rate within families, and perhaps even more so between families, we first proceeded to the characterization of samples before selecting specimens at stage T1, T2 and T22 as stage-specific samples. The embryos and T22 specimens were staged by developmental time and examination of behavior, morphological, and anatomical characteristics. For each time point, at list six specimens were observed using a stereo microscope (Zeiss Stemi 2000-C) under different magnification (10–50×), and images taken by means of a Megapixel digital camera connected to the microscope. The main characteristics defining each specific stage are reported in Table 2. The microscopic landmarks of these samples are presented in Additional file 22. Experimental protocols adopted were approved by the Review Committee for the Use of Animal Subjects of Shanghai Ocean University without requirement for particular permits. Sodium pentobarbital was employed to anesthetize larval samples, and attempts were made to minimize pain. Prior to RNA extraction, we removed the blastodisc cap from the surrounding yolk of the egg at either stage T1 and T2 to ensure that we were only examining embryonic RNA levels at these stages using the total yolk removal by bursting as described elsewhere [27]. Briefly, after isolation from chorion, the egg was brought to the surface of the liquid by sucking it slightly into a Pasteur pipette. Once in contact with the surface of the liquid, the yolk membrane was teared by the surface tension, which makes the yolk float out while the blastodisk rounds off and sinks to the bottom of the glass. Whole bodies of anesthetized larvae (T22) or isolated eggs (T1 and T2) were immediately placed in liquid nitrogen awaiting RNA extraction. Specimens were homogenized with Lysing Matrix D (Q-BioGene Inc., Carlsbad, CA, USA) for 40 s with velocity adjusted to 6 using the Fastprep FG120 instrument (Bio101, Thermo Savant Instruments). For each stage, total RNA was extracted from three specimens using Trizol (Invitrogen, CA, USA) in line with the manufacturer’s recommendations. The extracted RNA samples were quantified and purified using Bioanalyzer 2100 and RNA 6000 Nano LabChip Kit (Agilent, CA, USA) with RIN number >8.0. Samples of RNA extracted from specimens of each developmental stage were pooled together as one stage-specific sample. Afterward, the mRNA was isolated, fragmented into small pieces and reverse-transcribed into the cDNA libraries using the mRNA-Seq preparation kit (Illumina, San Diego, USA). Illumina Hiseq2000 platform was used for paired-end sequencing (2*100 bp) of cDNA libraries with normal insert size of 300 ± 50 bp.

**Table 2:** Main characteristics of studied specimens defining each developmental stage

Sample name	Developmental stage	Main morphological characteristics
T1	Big yolk plug	The formation of large yolk plug:
		About 32 h after fertilization, the embryo develops into yolk plug period: blastopore endodermal cells are still externally exposed at the vegetal pole, formation of a large yolk plug inserted in the germ ring, at this time the animal is very bright yellow and the plant polar pigmentation is very deep.
T2	Wide neural plate	Obvious wide neural plate divided into both internal and external parts: about 45 h after fertilization, there is apparition of an obvious wide neural plate; around the neural plate in the head region, there is a clear shaped horseshoe formation; the neural plate grows longitudinally and is divided into inner and outer parts; in the center of the neural plate grows a longitudinal neural groove.
T22	64-day-old fish with electrical receptors ganglion	Fully developed bone plate, 14–16 dorsal spines, 45–47 lateral bone plates, 7–9 abdominal bone plates. Presence of ampullary organs on the ventral face

### Sequencing data quality assessment and *de novo* assembly

Prior to assembly and mapping, we applied filters for quality control of sequenced reads. Trim Galore software was adopted for quality trimming of raw reads and dynamic removal of adapters and low-quality fragments.

High-quality sequence reads stemming from quality control analysis were *De novo* assembled into transcripts using the Trinity platform (http://trinityrnaseq.sf.net) including Inchworm, Chrysalis and Butterfly as independent modules following the protocol described elsewhere [19]. The evaluation of the efficacy of the assembly was performed taking into account the total number of transcripts, transcripts length distribution and N50 value. We calculated the N50 size according to the threshold of lengths of different transcripts and counted transcripts with lengths greater than or equal to the minimum threshold.

A unigene was defined as the longest transcript among the multitude of assembly transcripts isomers. The optimization of assembly led to different transcript isomers (isoforms) or paralogs. Each unigene (expressed in prefix comp + digital ID) corresponded to one or numerous transcripts isomers (comp*_c*_Seq*).

To discriminate between valid transcript sequences and incorrectly assembled sequences, we used TransDecoder program integrated in Trinity software (log likelihood ratio based on the ratio of coding to noncoding sequences) to extract open reading frames (ORFs) and predict potential protein coding domain sequences (CDS) according to Markov model principle. CDS were translated into amino acid sequences according to the standard codon table in order to obtain potential protein sequences coded by the transcripts.

### Functional annotation of unigenes

*De novo* functional annotation of *A.baeri* transcriptome was obtained by similarity search against UniProt protein databases (Swissprot or Tremble) using BLASTX. Alignments with an E-value cut off of 1E-3 were considered significant and gene annotation information was assigned to transcripts based on the highest BLAST hit. The Blast2GO suite [28] was used for the Gene Ontology (GO) annotation of unigenes. Annotation via Blast2GO was done by first searching for matches to the Uniprot databases, then mapping the BLAST results to the GO database and finally retrieving GO annotation information corresponding to transcripts with BLAST hits. The WEGO software was applied for classifying and counting GO classes. Additionally, the KEGG annotation of unigenes was achieved using the online KEGG database (http://www.genome.jp/kegg/). Using the HMM algorithm, Interproscan (http://www.ebi.ac.uk/InterProScan/), including PRINTS, SMART, Pfam, Coils, SUPERFAMILY, Gene3D, ProSiteProfiles, Hamap, ProSitePatterns, TIGRFAM and PIRSF databases, was performed for searching for protein domains.

### Unigenes abundance estimation

To compute abundance estimates of transcripts, the original reads were firstly aligned to the Trinity transcripts. Subsequently, the RNA-Seq by Expectation-Maximization (RSEM) software [29] (default parameter Settings) was implemented to determine the expression levels of transcripts or corresponding unigenes in FPKM (expected number of fragments per kilobase of transcript sequence per millions base pairs sequenced) units.

### Identification and functional enrichment analysis of differentially expressed genes

The Bioconductor tool edgeR [15] (*p*-value < 0.001, FDR <0.001) was exerted for screening differentially expressed genes (DEGs) by pairwise comparison between samples.

Functional enrichment analysis of DEGs was performed by mapping to terms in GO and KEGG databases. For the GO enrichment analysis, the total gene set was considered as a background list and differential genes list as the screened list obtained from the background list. The hyper-geometric test was used to calculate the *P*-value of significant or non-significant GO-terms of DEGs. FDR was obtained after correction for multiple testing of *p*-values using the Benjamini and Hochberg procedure. The hyper-geometric formula for computing *p*-value was as follows:

$$ \mathrm{P}\kern0.5em =\kern0.5em {\displaystyle \sum_{\mathrm{i}=0}^{\mathrm{m}-1}\frac{\left(\begin{array}{c}\hfill M\hfill \\ {}\hfill i\hfill \end{array}\right)\left(\begin{array}{c}\hfill N-M\hfill \\ {}\hfill n-i\hfill \end{array}\right)}{\left(\begin{array}{c}\hfill M\hfill \\ {}\hfill n\hfill \end{array}\right)}} $$

Note: N stands for the number of genes with GO annotation among all genes, n is the number of DEGs in N, M is the number of all genes that are annotated to a certain GO term; and m is the number of DEGs in M.

The method used for KEGG enrichment analysis was similar to that used in the GO enrichment analysis. Here, N is the number of genes with a KEGG annotation, n is the number of DEGs in N, M is the number of genes assigned to a specific pathway, and m is the number of DEGs in M. Pathways with a *P*-value < 0.05 were defined as significantly enriched pathways.

### Quantitative real-time PCR analysis

Quantitative real-time PCR (qRT-PCR) was performed to validate the RNA-seq data. The pool of RNA used for generating our RNA-seq libraries were reverse transcribed into cDNA using the PrimeScript™ RT reagent Kit (TaKaRa, China) following to the manufacturer’s instructions with random hexamer primers which were designed by means of Primer Premier v5.0 software (Premier Biosoft, USA). qRT-PCR was achieved with the QuantiFast SYBR Green PCR Kit (Qiagen, Germany) using a 20 μl reaction volume according to the manufacturer’s protocol. The fluorescence intensity was determined by CFX96™ Real-Time System (Bio-Rad, USA). Each reaction was performed in triplicates. Candidate gene expression levels were normalized using the 15ACTB gene as endogenous control. 15ACTB was chosen based on the results of preliminary experiments regarding the selection of a stable housekeeping gene among 15ACTB, GAPDH and α-tubulin using geNorm [30], NormFinder [31] and BestKeeper [32] algorithms. All PCR primers used in this study are summarized in Table 3. The relative abundance of transcript was estimated using the comparative Cт (ΔΔCт) method. The data were shown as the mean ± SD and one-way ANOVA with Bofferoni posttest was used for intergroup comparison of gene expression.

**Table 3:** List of primers designed for qRT-PCR validation of RNA-seq data

Unigene Id	Gene name	Primers
comp134704_c0_seq1	Sex determining	F:5ʹAGTCCTGACGCTGGGTATGC 3ʹ
	Region Y-Box 17 (sox17)	R:5ʹGTCGCCGTATCCGAGGTTC 3ʹ
comp135742_c0_seq2	SIX homeobox 3a (six3a)	F:5ʹTTATCCCTCCCATTTCTTCCTG 3ʹ
		R:5ʹGAGAAGTTGAGGGTCGGTAGCT 3ʹ
comp137301_c0_seq3	homeobox D10 (HOXD10)	F:5ʹGCTCTCCTGCTGCTAATACCTT 3ʹ
		R:5ʹCGGCAGTAGTCCACAGGTTT 3ʹ
comp141980_c0_seq1	wingless-type MMTV integration site family, member 11B (wnt11b)	F:5ʹGTAGCCTCGGCCACAGCA 3ʹ
		R:5ʹGCAGACCTCAAGTCCAAATACCT 3ʹ
comp128400_c0_seq2	EYA transcriptional coactivator and phosphatase 3 (eya3)	F:5ʹACCCACCTGGCTGCGAGT 3ʹ
		R:5ʹGGACGAAGCCCTGGAACTG 3ʹ
comp118626_c0_seq1	actin, beta (Actb)	F:5ʹAGGTCCTTACGGATGTCAACG 3ʹ
		R:5ʹGCGTTTCAGGTGCCCAGA 3ʹ

## Additional files

Additional file 1: Result file of the *de novo* assembly of sequenced reads into unigenes. (XLSX 41231 kb) Additional file 2: N50 statistics of the sequencing data. (XLS 11 kb) Additional file 3: Results file of the best matches of unigenes obtained from the alignment against Uniprot database. (XLS 9825 kb) Additional file 4: Statistics of hits of *A. baeri*’s sequences to species in the Uniprot database. (XLS 33 kb) Additional file 5: Unigenes GO annotation results file. (XLS 107520 kb) Additional file 6: Unigenes KEGG pathway annotation results file. (XLS 646 kb) Additional file 7: Unigenes interpro annotation results file. (XLS 35 kb) Additional file 8: Interproscan domain statistics. (XLS 250 kb) Additional file 9: Expression level of unigenes estimated by RSEM in FPKM units. (XLSX 1107 kb) Additional file 10: EdgeR output files for pairwise comparison of samples for identification of DEGs. (XLSX 684 kb) Additional file 11: List of significant differentially expressed unigenes for each pair of EdgeR comparison of each pair of samples. (XLSX 2223 kb) Additional file 12: Significant subclusters obtained from the hierarchical clustering of DEGs during development. (PDF 73 kb) Additional file 13: Significantly enriched GO-terms obtained from the GO enrichment analysis of DEGs between T1-T2. (XLSX 26 kb) Additional file 14: Significantly enriched pathways obtained from the KEGG pathway enrichment analysis of DEGs between T1 and T2. (XLSX 11 kb) Additional file 15: GO enrichment analysis of DEGs screened between T1 and T22. (XLSX 147 kb) Additional file 16: KEGG pathway enrichment analysis of DEGs screened between T1 and T22. (XLSX 12 kb) Additional file 17: GO enrichment analysis of DEGs screened between T2 and T22. (XLSX 93 kb) Additional file 18: KEGG pathway enrichment analysis of DEGs screened between T2 and T22. (XLSX 13 kb) Additional file 19: List of unigenes associated with morphogenesis as screened from GO enrichment file and their expression profile. (XLSX 49 kb) Additional file 20: List of the set of development regulatory unigenes screened from the GO enrichment file and their expression dynamics. (XLSX 133 kb) Additional file 21: qRT-PCR validation of *A. baeri* RNA sequencing data based on selected candidate genes. The pool of RNA used for generating the RNA-seq libraries was used for determining the expression of candidate unigenes using a Biorad CFX96™ Real-Time System. (A) FPKM levels obtained from sequencing data. (B) mRNA expression levels obtained from the qRT-PCR experiments relative to 15ACTB gene expression. Errors bar represent the standard deviation calculated from the mean of three independent measures. * indicates *P* < 0.05 compared with T1 and shows # *P* < 0.05 relative to T1 and T2. No error bar was indicated for the RNA-seq data due to the fact that these data were unreplicated. (TIF 722 kb) Additional file 22: Microscopic images of fish samples. (PDF 63 kb)
