# Supplementary material for: RNA-sequencing of the sturgeon Acipenser baeri provides insights into expression dynamics of morphogenic differentiation and developmental regulatory genes in early versus late developmental stages
Source: BMC Genomics. 2016 Aug 8;17:564. doi: 10.1186/s12864-016-2839-3 (PMC4977659; doi:10.1186/s12864-016-2839-3)
Supplement: Additional file 12: — Significant subclusters obtained from the hierarchical clustering of DEGs during development. (PDF 73 kb) [file 12864_2016_2839_MOESM12_ESM.pdf]

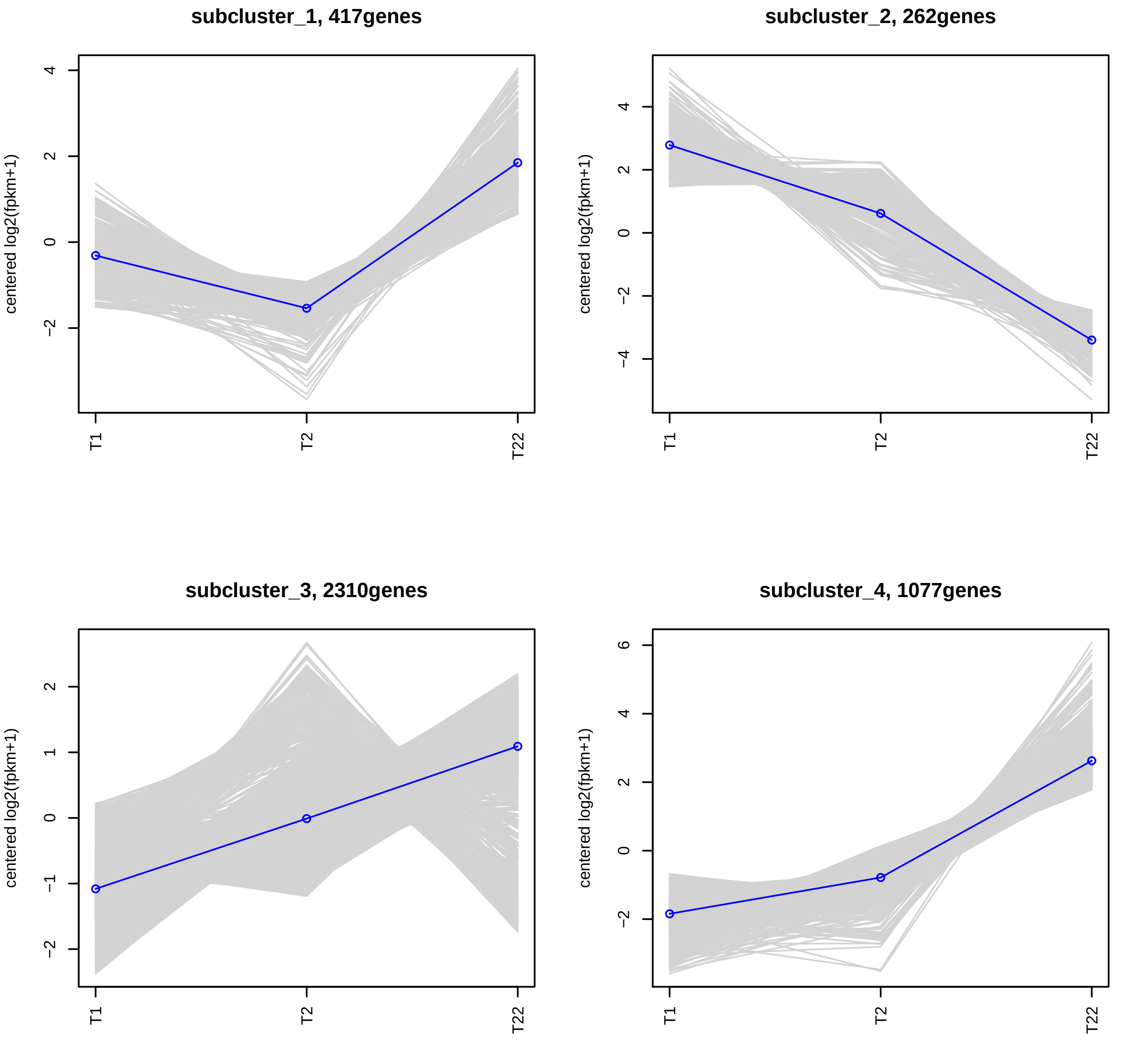
subcluster_1, 417genes
subcluster_2, 262genes
subcluster_3, 2310genes
subcluster_4, 1077genes
centered log2(fpkm+1)
T1
T2
T22
4
2
0
−2
−4
6
1
−1

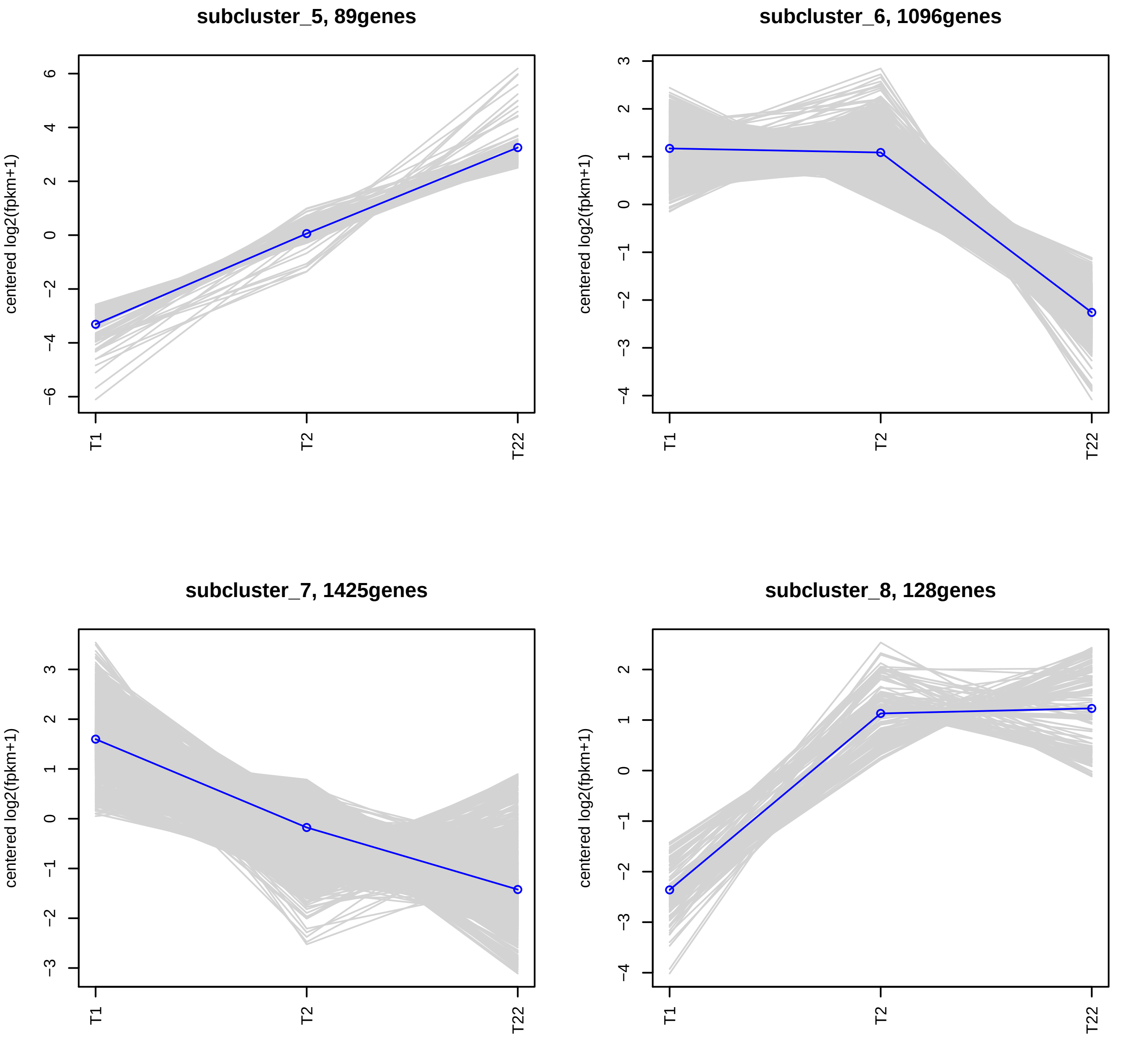

subcluster_5, 89genes
subcluster_6, 1096genes
subcluster_7, 1425genes
subcluster_8, 128genes
centered log2(fpkm+1)
T1
T2
T22

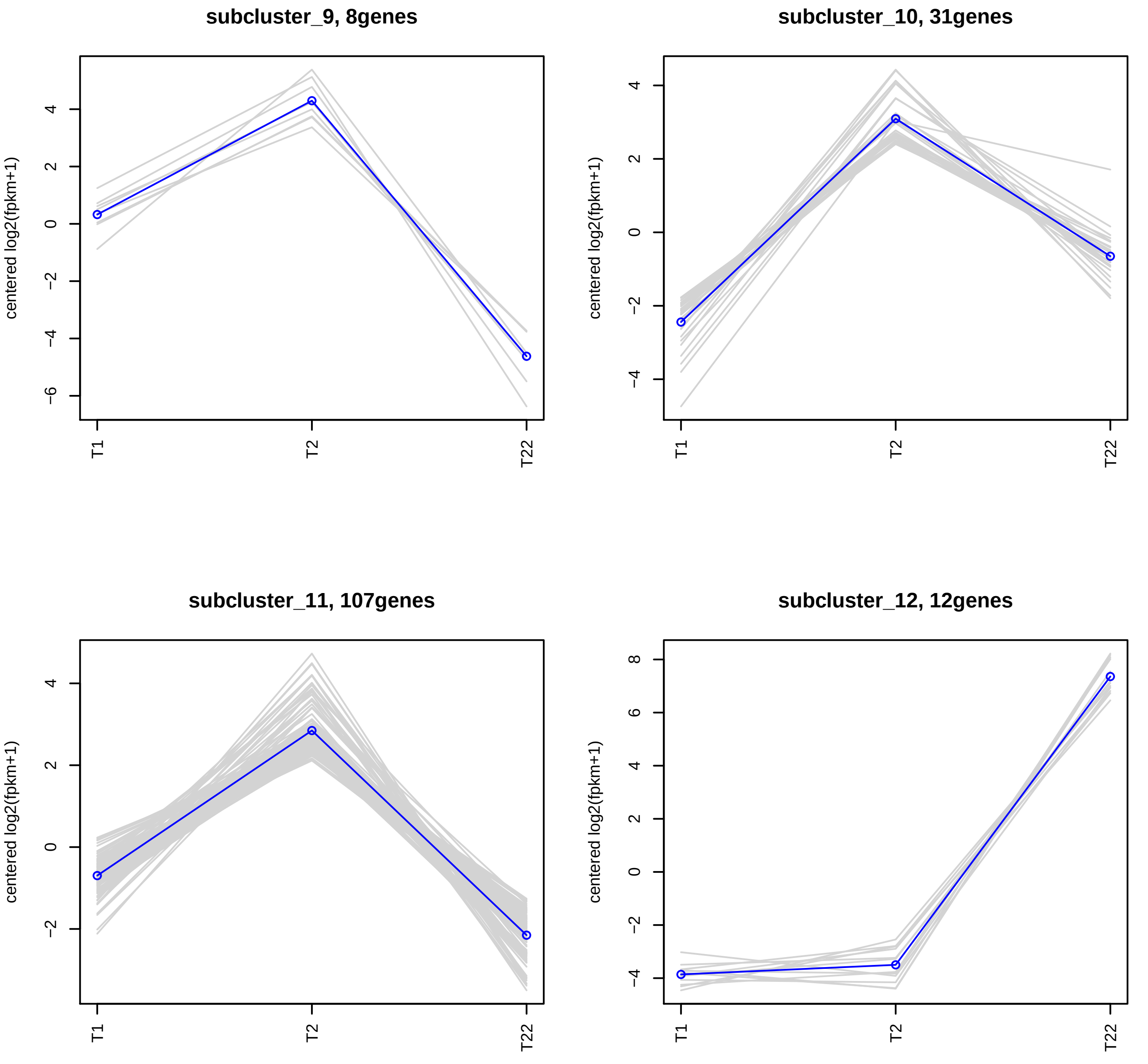
subcluster_9, 8genes
centered log2(fpkm+1)
4
2
0
−2
−4
−6
T1
T2
T22
subcluster_10, 31genes
centered log2(fpkm+1)
4
2
0
−2
−4
T1
T2
T22
subcluster_11, 107genes
centered log2(fpkm+1)
4
2
0
−2
T1
T2
T22
subcluster_12, 12genes
centered log2(fpkm+1)
8
6
4
2
0
−2
−4
T1
T2
T22
