# Supplementary figures and images for: RNA-sequencing of the sturgeon Acipenser baeri provides insights into expression dynamics of morphogenic differentiation and developmental regulatory genes in early versus late developmental stages
Source: BMC Genomics. 2016 Aug 8;17:564. doi: 10.1186/s12864-016-2839-3 (PMC4977659; doi:10.1186/s12864-016-2839-3)

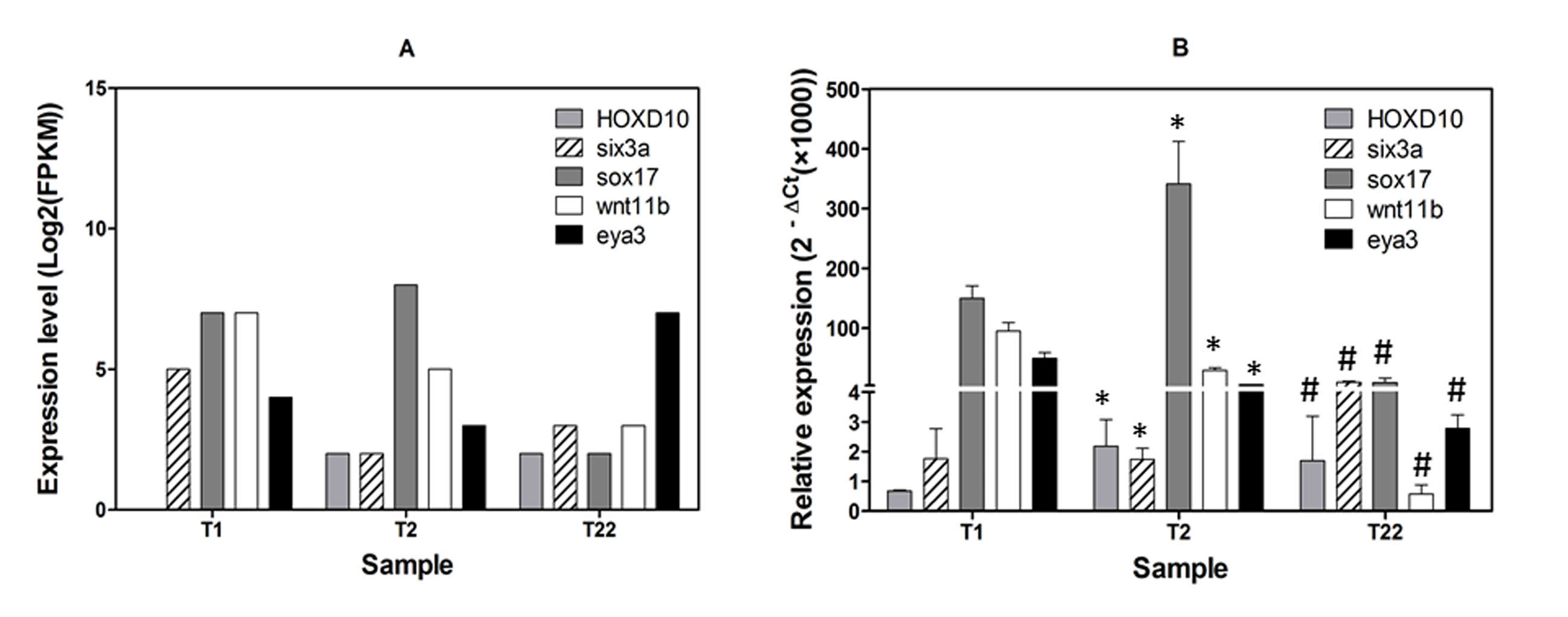

Supplement: Additional file 21: — qRT-PCR validation of A. baeri RNA sequencing data based on selected candidate genes. The pool of RNA used for generating the RNA-seq libraries was used for determining the expression of candidate unigenes using a Biorad CFX96™ Real-Time System. (A) FPKM levels obtained from sequencing data. (B) mRNA expression levels obtained from the qRT-PCR experiments relative to 15ACTB gene expression. Errors bar represent the standard deviation calculated from the mean of three independent measures. * indicates P < 0.05 compared with T1 and shows # P < 0.05 relative to T1 and T2. No error bar was indicated for the RNA-seq data due to the fact that these data were unreplicated. (TIF 722 kb) [file 12864_2016_2839_MOESM21_ESM.tif]

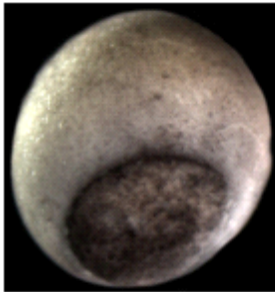

T1

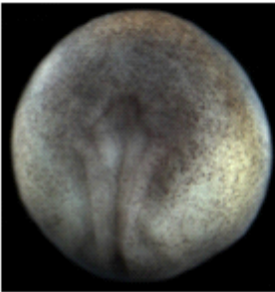

T2

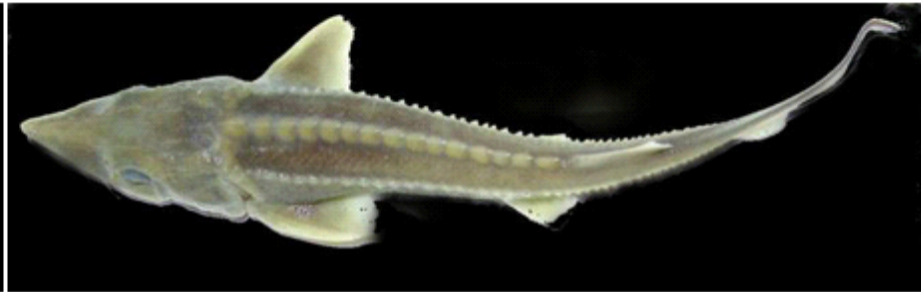

T22

Supplement: Additional file 22: — Microscopic images of fish samples. (PDF 63 kb) [file 12864_2016_2839_MOESM22_ESM.pdf]
